# Neurodegeneration: Keeping ATF4 on a Tight Leash

**DOI:** 10.3389/fncel.2017.00410

**Published:** 2017-12-15

**Authors:** Priyamvada M. Pitale, Oleg Gorbatyuk, Marina Gorbatyuk

**Affiliations:** ^1^Department of Optometry and Vision Science, School of Optometry, University of Alabama at Birmingham, Birmingham, AL, United States; ^2^Department of Neurology, Center for Neurodegeneration and Experimental Therapeutics, University of Alabama at Birmingham, Birmingham, AL, United States

**Keywords:** activating transcription factor 4, ER stress response, unfolded protein response (UPR), neurodegenerative diseases, retinal diseases, neurons, photoreceptor cells, vertebrate

## Abstract

Activation of the endoplasmic reticulum (ER) stress and ER stress response, also known as the unfolded protein response (UPR), is common to various degenerative disorders. Therefore, signaling components of the UPR are currently emerging as potential targets for intervention and treatment of human diseases. One UPR signaling member, activating transcription factor 4 (ATF4), has been found up-regulated in many pathological conditions, pointing to therapeutic potential in targeting its expression. In cells, ATF4 governs multiple signaling pathways, including autophagy, oxidative stress, inflammation, and translation, suggesting a multifaceted role of ATF4 in the progression of various pathologies. However, ATF4 has been shown to trigger both pro-survival and pro-death pathways, and this, perhaps, can explain the contradictory opinions in current literature regarding targeting ATF4 for clinical application. In this review, we summarized recent published studies from our labs and others that focus on the therapeutic potential of the strategy controlling ATF4 expression in different retinal and neurodegenerative disorders.

## Introduction

**Figure 1:**
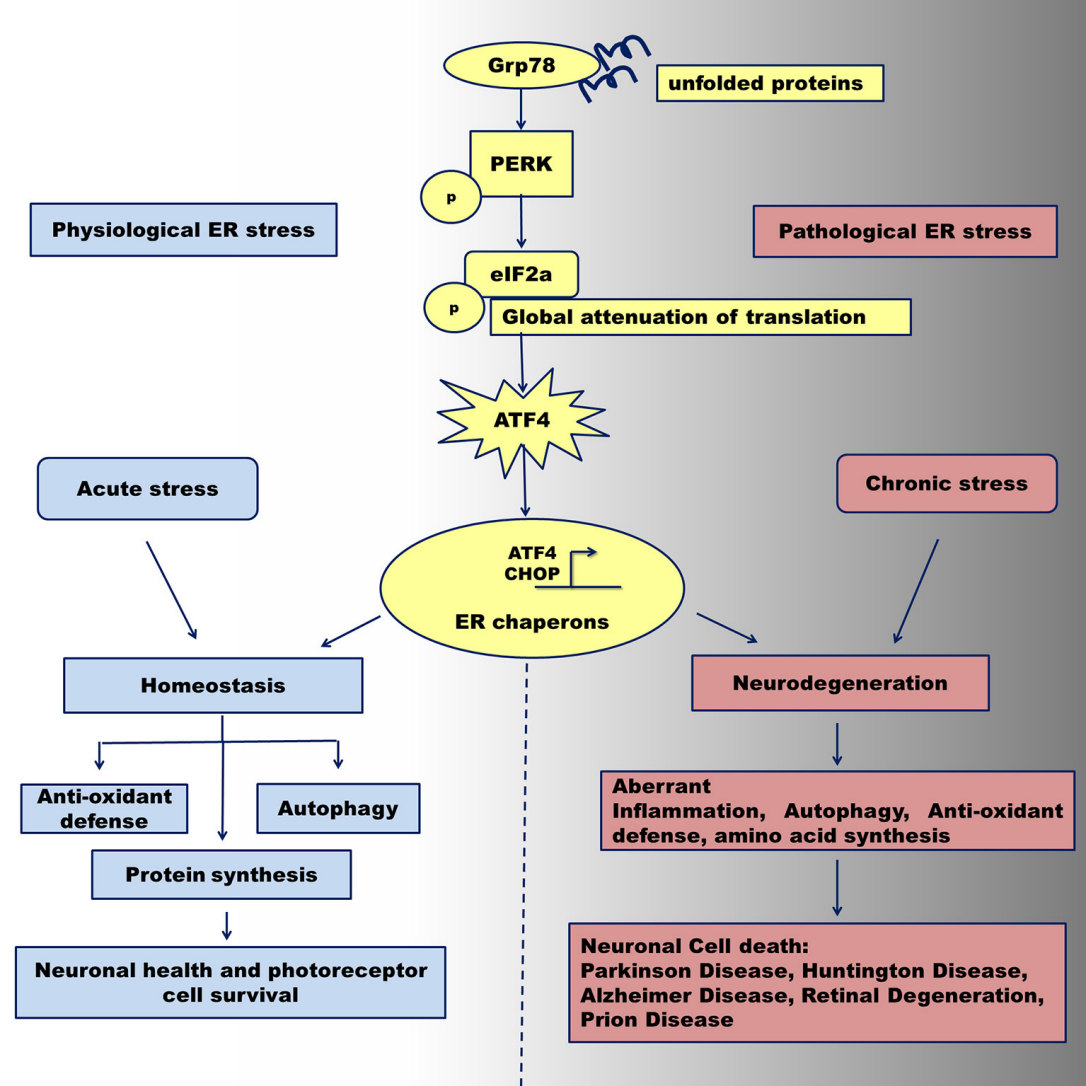
Activation of the PERK UPR signaling under physiological and pathological ER stress conditions. Abnormally folded proteins within the ER are sensed by GRP78 chaperone that in turn activates PERK pathway program. Under acute ER stress, the PERK kinase activates downstream mediator, phosphorylated eIF2a leading to up-regulation of ATF4. The latest together with CHOP protein activates transcription of ER stress chaperones that can regulate the transition from adaptation and neuronal cell survival via temporal translation inhibition leading to reestablishing of cellular homeostasis. After prolonged pathological ER stress, ATF4 can regulate the transition from adaptation/survival in neuronal cells to a pro-apoptotic phase via provoking aberrant autophagy signaling, translational program, and inflammatory response. Sustained malfunction of these cellular signaling may contribute to neuronal cells death through activation of apoptosis and leads to neurodegeneration seen in Parkinson disease, Alzheimer Disease, Huntington Diseases, Prion Disease and Retinal Degeneration.

Mammalian cells activate the unfolded protein response (UPR) in response to various internal and external cellular stimuli that disturb the balance in the endoplasmic reticulum (ER). These stimuli include, but are not limited to, accumulation of misfolded proteins, glucose deprivation (Roth et al., 2010; Csala et al., 2012), calcium dysfunction (Krebs et al., 2011; Shinde et al., 2016), hypoxia (Zheng et al., 2012), inflammation (Wang et al., 2016), redox potential changes (Hagiwara and Nagata, 2012), and mechanical stress (Husa et al., 2013). The normal physiological process involves activation of a signaling cascade, mediated by dissociation of the stress sensors protein kinase R (PKR)-like endoplasmic reticulum kinase (PERK), inositol requiring enzyme I (IRE-1), and activating transcription factor 6 (ATF 6) from glucose regulated protein 78 (Grp78), a key regulator of the UPR (Patil and Walter, 2001). This activation results in adjustment of the ER capacity to assist protein in the folding, activation of the ER-associated degradation (ERAD) pathway for degradation of abnormal proteins, and re-establishment of cellular homeostasis. However, a stimulus of long-lasting duration may cause a chronic ER stress. In this case, the cell experiences a “pathological” UPR that triggers programmed cell death, usually through sustained activation of the PERK UPR arm and pro-apoptotic components of cellular signaling (Figure 1; Woehlbier and Hetz, 2011). Detection of the physiological UPR could be challenging in cells due to the dynamics of downstream molecular events and quick equilibration of cellular homeostasis. By contrast, pathological UPR could be traced by accumulation of the UPR hallmark proteins and histological observation of consequent cell death (Hiramatsu et al., 2011). Recognition of a molecular switch operating between cell life and death decisions is perhaps a cornerstone of contemporary cell physiology and potentially will be a focus of future investigations.

At the molecular level, a cell under chronic ER stress continues to activate the PERK signaling pathway (Woehlbier and Hetz, 2011). Following transduction of information regarding the protein capacity of the ER, the IRE1 pathway is turned off (Woehlbier and Hetz, 2011). At the same time, the activation of the PERK downstream mediator activating transcription factor 4 (ATF4) induces the expression of a transcriptional factor C/EBP Homologous Protein (CHOP), which is, in turn, responsible for the activation of apoptosis through, Bcl-2-like protein 11 (BIM), p53 upregulated modulator of apoptosis (PUMA), and Noxa up-regulation (Huang et al., 2015). Therefore, the expression, activation, and duration of ATF4 activity could be an important parameter that determines cell fate. In this review, we summarized the latest discoveries on the role of the ATF4 protein in various retinal and neurodegenerative diseases.

## ATF4: expression and post-translational modification

ATF4 is a 351 amino acid cAMP-response element binding protein that belongs to the cAMP response element-binding protein (CREB)-2 family of proteins (Vattem and Wek, 2004). The protein is structured into several domains that are essential for ATF4 function and degradation. The N-terminal of ATF4 contains a p300/CBP associated factor (PCAF) domain for p300 interaction, a leucine zing finger region II for the binding of neuronal cell death putative kinase, and a prolyl-4 hydroxylase domain (Hai and Curran, 1991; Karin and Smeal, 1992; Chérasse et al., 2007; Chan et al., 2013). The leucine zipper is essential for the dimerization of two DNA binding regions and for recognition of 5′-TGACGTCA-3′ DNA sequence within promoters(Vincent and Struhl, 1992). Two other domains, the oxygen dependent degradation (ODDD) domain and βTrCP motif, are involved in ATF4 degradation.

At the C-terminal, ATF4 contains the basic/leucine zipper domain (bZIP domain) that serves as a DNA binding sequence for interaction with abraxas brother 1 (Abro1), CCAAT/enhancer binding protein β (C/EBPβ), factor-inhibiting ATF4-mediated transcription (FIAT), AT-rich sequence-binding protein 2 (SATB2), death-associated protein Ser/Thr kinase 3 (DAPK3), and nuclear factor (erythroid- derived 2)-like 2 (Nrf2) (He et al., 2001). The ATF4 mRNA is transcribed ubiquitously at low levels, but its protein expression depends on various stress conditions, such as hypoxia, anoxia, and lack of nutrition, as well as glucose deprivation. The mouse ATF4 mRNA contains two upstream open reading frames (uORFs): the uORF1 and uORF2 located at 5′ of coding sequence. ATF4 expression involves a differential contribution of each upstream ORF(Vattem and Wek, 2004).

The uORF1 is a positive acting element that facilitates ribosome scanning (Wek et al., 2006), whereas the uORF2 acts as an inhibitory element that blocks the ATF4 expression that promotes ribosome scanning and re-initiation at the ATF4 coding sequence. As a part of a translational complex, the eukaryotic initiation factor 2 alpha (eIF2a) forms a ternary structure with GTP and Met-tRNA to bind the 40S ribosomal subunit and initiate translation (Kilberg et al., 2009). Under non-stress conditions, when eIF2a-GTP is plentiful due to the absence of stress, the complex binds a ribosome, GTP is hydrolyzed, and eIF2a-GDP is released from the complex for further association with eIF2b, an eIF2a GDP-GTP exchanger. This results in re-initiation of downstream ribosome scanning at the next uORF2 (Vattem and Wek, 2004; Wek et al., 2006). After translation of uORF2, ribosomes dissociate from the ATF4 mRNA. This leads to a reduction in expression of the ATF4-coding region and a weak ATF4 expression (Vattem and Wek, 2004). Under stress conditions, eIF2a is phosphorylated by four different kinases [PERK, protein kinase R(PKR), general control nonderepressible 2 (GCN2), and heme-regulated eIF2α kinase (HRI) kinases; (Vattem and Wek, 2004; Wek et al., 2006)], and this phosphorylation inhibits eIF2b-mediated eIF2a GDP-GTP exchange and represses global protein synthesis (Vattem and Wek, 2004). Meanwhile, eIF2a-GTP levels are lowered, and this deficit causes further ribosome scanning to be performed more carefully through negative-acting ORF2 positions at the initiation of the ATF4-coding region (Vattem and Wek, 2004). Therefore, translation of ATF4 is significantly enhanced in response to cellular stress.

In addition to the bZip domain, the ATF4 protein contains multiple sites for post-translational modification, including phosphorylation sites and sites for ubiquitination, SUMOylation, and acetylation. Protein kinase A (Karpinski et al., 1992), ribosomal S6 kinase 2 (Li et al., 2014), protein kinase CK2 (Manni et al., 2012), and RET tyrosine kinase (Bagheri-Yarmand et al., 2015) are all known to phosphorylate ATF4. At many positions, phosphorylation leads to ATF4 degradation (Lassot et al., 2001). ATF4 degradation also relies on a phosphorylation-dependent interaction with the SCF (betaTrCP) ubiquitin ligase (Lassot et al., 2001). Along with phosphorylation, another mechanism that controls the ATF4 activity is the binding of p300, NAD-dependent deacetylase sirtuin-1 (SIRT1), and FIAT. Histone acetyltransferase p300 binds to the N-terminal of ATF4, thereby stabilizing and enhancing ATF4 transcriptional activity (Lassot et al., 2005). Conversely, FIAT protein blocks the ATF4 activity by binding to a leucine zipper motif and preventing ATF4 from further associations. This motif is directly responsible for ATF4 inhibition when FIAT is over-expressed in cells (St-Arnaud and Elchaarani, 2007; Yu et al., 2009; St-Arnaud et al., 2010). In addition, NAD^+^-dependent deacetylase SIRT1 also downregulates ATF4 synthesis during proteasome inhibition in cells (Woo et al., 2013).

## ATF4 activates pro-survival and pro-death signaling during neurodegeneration

The activity of ATF4 is associated with its cellular localization. Under conditions of ER stress, ATF4 migrates to the nucleus, using the nucleus targeting KKLKK signal (amino acids 280 to 284) located within the basic region of the ATF4 gene. In the nucleus, ATF4 binds targeted DNAs and regulates their transcription (Han et al., 2013). The C/EBP homologous protein (CHOP, also known as GADD153) is one of the targeted genes. Together with ATF4, this protein regulates the expression of various cellular genes (Han et al., 2013). The list of shared targets includes 218 proteins. CHOP and ATF4 regulate multiple cellular processes, including the cellular response to ER stress (chaperones), protein biosynthesis, translation, and amino-tRNA synthetase activity. In addition, ATF4 controls the expression of 254 genes independently of CHOP (Han et al., 2013). These genes mostly govern cellular metabolism, including amino acid biosynthesis and transporter activity.

Multiple studies have identified ATF4 downstream targeted genes, but very few *in vivo* experiments have investigated up- and down-regulation of ATF4 in the brain or in eye tissues. We recently over-expressed ATF4 in the mouse retinal and rat dopamine nigral neurons (Bhootada et al., 2016; Gully et al., 2016). To do this, we leveraged the ability of the adeno-associated viruses with serotype 5 to deliver ATF4 cDNA, and we efficiently transduced the cDNA in photoreceptors of the retina or dopaminergic neurons of the substantia nigra pars compacta (SNc). We revealed that a 2.3-fold increase in ATF4 triggers retinal degeneration in wild type mice and mimics conditions with ATF4 over-expression in transgenic T17M rhodopsin retinas. Analysis of ERG recordings demonstrated a significant reduction in the functional tests in both types of retina (Bhootada et al., 2016). The functional loss in the retina was associated with a higher rate of photoreceptor cell death in mutant retinas over-expressing ATF4. Therefore, both the wild type and the mutant retinas experienced activation of apoptotic pathway.

We also conducted a second study, in which we investigated rats with a 3.2-fold increase in ATF4 in the SNc. These rats showed a loss of TH-positive cells when compared to control rats given AAV5-GFP injections (Gully et al., 2016). Cell loss was associated with a drop in the dopamine level and an increased activation of caspase-3/7. Taken together, our data on retinal and nigral neurons have demonstrated that over-expression of ATF4 results in progressive neurodegeneration. This discovery led to the next intriguing question regarding the cause of death of the neuronal cells over-expressing ATF4. We detected activation of caspase 3/7 in both studies, but we have not yet identified a direct molecular path that leads to neuronal cell death. The ATF4 and the ATF4 → CHOP signaling together can modulate the expression of 575 genes, which could eventually lead to neuronal death in these tissues (Han et al., 2013). Therefore, conducting further experiments with ATF4 titration and CHOP knockdown in these cells would be interesting in order to obtain a precise answer to this question.

## ATF4 and retinal degenerative diseases

Retinal degeneration is a progressive deterioration of retinal cells that eventually leads to their demise. The group of retinal degenerative disorders includes inherited, trauma-associated, diabetic, and age- related retinal degenerations. The cellular mechanism of retinopathies is complex and is often linked to multiple molecular markers of autophagy, oxidative stress, and inflammation. The UPR PERK → p- eIF2a → ATF4 pathway seems to play a significant role in photoreceptor deterioration in mice expressing aberrant proteins. Thus, we and other investigators have recently proposed that the UPR contributes markedly to retinal pathogenesis under various degenerative conditions (Gorbatyuk and Gorbatyuk, 2013; Zhang et al., 2014; Hiramatsu et al., 2015; Karthikeyan et al., 2017). Some investigators have suggested that “ER stress is a general upstream mechanism for neurodegeneration” and that “targeting ER stress molecules is a promising therapeutic strategy for neuroprotection (Huang et al., 2017).”

Research conducted over the past 5 years has identified ATF4 as one of the cellular markers that is elevated by various stress conditions in the retina. We have demonstrated that mice expressing mutant and truncated rhodopsins show dramatically elevated ATF4 levels (Kunte et al., 2012; Rana et al., 2014; Bhootada et al., 2016), while other investigators have detected ATF4 upregulation in different models of inherited retinal degeneration (Comitato et al., 2016; Lobo et al., 2016; Ooe et al., 2017). ATF4 regulates inflammatory signaling by governing the expression of multiple inflammatory genes. For example, in wild type mice, we found that ATF4 over-expression significantly increased the production of pro-inflammatory IL-1β (Rana et al., 2014). Huang et al. reported that ATF4 is a novel regulator of monocyte chemoattractant protein-1 (MCP-1) (Huang et al., 2015). These cytokines are all overproduced in the mouse retina during retinal degeneration. Therefore, not surprisingly, ATF4 downregulation in the T17M rhodopsin retina diminishes the ER stress response and prevents of loss of retinal function and photoreceptor cell death (Bhootada et al., 2016).

In degenerating retinas, enhanced ATF4 expression could be provoked by excessive light. Kuse et al. reported that blue light triggers photoreceptor cell death through activation of an ER stress response and ATF4 over-production (Kuse et al., 2014). This group subsequently demonstrated that light-induced S- opsin aggregation could be responsible for the activation of ATF4 (Ooe et al., 2017). Taken together, these findings would imply that mislocalization of opsin in the photoreceptors, on its own, is capable of triggering the ER stress response through activation of ATF4 signaling.

ATF4 plays a critical role in the progression of diabetic retinopathy (DR). Studies of proliferative DR have proposed ATF4 as one of the bio-markers of DR and suggest that it may represent a new therapeutic target for proliferative DR (Wang et al., 2017). Support for this proposal comes from a mouse model of type 1 diabetes with an activated ER stress response that inhibits ATF4 activity; these mice show a marked attenuation of the high–glucose-induced production of Intercellular Adhesion Molecule (iCAM), Tumor necrosis factor (TNFa), and vascular endothelial growth factor (VEGF) and a significant overall amelioration of retinal inflammation (Chen et al., 2012). Similarly, ATF4 over-expression triggers an inflammatory response in endothelial cells through activation of the Signal transducer and activator of transcription 3 (STAT3) pathway (Chen et al., 2012). This could explain why T17M rhodopsin mice with inherited retinal degeneration experience a preservation of retinal function and photoreceptor cell death when their retina is deficient in TNFa, a downstream ATF4 target (Rana et al., 2017).

Another hallmark of proliferative DR, and of the wet form of age-related macular degeneration, is angiogenesis. Our study of the hypoxic retina demonstrated that ATF4 deficiency could alleviate hypoxia-driven neovascularization in mice (Wang et al., 2013). Other investigators have also confirmed that ATF4 is a powerful promoter of angiogenesis (Liu et al., 2015; Chen et al., 2017). Taken together, these studies emphasize the need for further studies to determine the role of ATF4 in various neovascularization conditions.

## ATF4 and CNS neurodegenerative diseases

Neurodegenerative diseases, including Alzheimer's disease (AD), Parkinson's disease (PD), Huntington's disease (HD), and prion disease, are characterized by the slow and progressive loss of neural cells and subsequent dysfunction of the nervous system. Accumulation of misfolded proteins has been proposed as a common link in many neurodegenerative disorders (Li et al., 2013; Roussel et al., 2013; Scheper and Hoozemans, 2015; Remondelli and Renna, 2017). Thus, a study of neurodegeneration in a mouse model of AD revealed an activation of ATF4-mediated intra-axonal translation and identified ATF4 as a mediator of the spread of AD neurodegeneration (Baleriola et al., 2014). The AD brain (Ohno, 2014) and the brain of this AD-like mouse model (Jankowsky et al., 2004) both showed significant upregulation of the protein level of ATF4. This is perhaps associated with the fact that ATF4 upregulation “may not only act as the downstream effector of Aβ but also as an upstream initiator for the memory deficit and pathological hallmarks in AD (Wei et al., 2015).” An association is possible between ATF4 elevation and increased phosphorylation of tau, through Glycogen Synthase Kinase 3 (GSK-3) and Protein phosphatase 1 (PP1) kinases, and this could give rise to neuronal damage.

Sbodio et al., in their studies on the pathogenesis of HD, have demonstrated that dysfunction of ATF4, as a master regulator of amino acid homeostasis in cells, significantly contributes to molecular neurodegeneration. They postulated a disruption of cysteine biosynthesis in cells with polyglutamine repeats due to low activity of the cystathionine γ-lyase enzyme, which is due, in turn, to low ATF4 activity (Sbodio et al., 2016). They proposed that, under conditions where the oxidative stress persists or exceeds a certain level in a cell, ATF4 loses its ability to restore cellular responses and homeostasis. This implies that patients with HD might benefit from restoration of ATF4 activity that regulates a diverse spectrum of amino acid metabolism.

The cellular mechanism of PD pathogenesis involves the aggregation of α-synuclein (αS) in the neurons in the form of Lewy bodies and substantial loss of nigral dopaminergic neurons in the SNc. Studies in a PD transgenic mouse model expressing αS120 (Bellucci et al., 2011) and in patient brain biopsies (Sun et al., 2013) have reported elevated levels of ATF4 in the SNc. For example, Sun et al. demonstrated that 50% of all studied human PD brain sections contained nearly 80% neuromelanin-positive neurons that showed strong ATF4 immunostaining (Sun et al., 2013). The remaining 50% of the sections showed ATF4 staining compatible with that observed in control brains. The authors proposed that the mean duration of the disease significantly affected ATF4 expression and that a longer PD progression gave rise to a higher ATF4 expression.

The role of ATF4 has been further explored by Imai et al. and Bouman et al., who proposed that the PD-like progression in mice could involve the binding of activated ATF4 to the E3 ligase parkin promoter (Imai et al., 2000, 2001) and the mediation of parkin expression in response to ER and mitochondrial stress (Bouman et al., 2011). The authors suggest that increased expression of parkin is beneficial in the context of protection of cells from ER stress and mitochondrial damage, so that ATF4 may play a cytoprotective role during PD development. Notably, in our previous work, we showed the association between experimental PD progression and ATF4 protein level (Gorbatyuk et al., 2012). We found that recombinant adeno-associated virus r(AAV)- mediated overexpression of human αS results in a seven-fold induction of the ATF4 protein. Our study of α-synucleopathy progression in rats demonstrated that the elevation of ATF4 occurring during PD progression is associated with a loss of TH positive cells, a reduced dopamine level, and an increased behavior deficit (Gorbatyuk et al., 2012). We therefore hypothesized that elevation of ATF4 could have a deleterious role in the brain in patients with PD. To this end, we used AAV to overexpress ATF4 in the SNc of the rat and compare human αS over-expression. We found that the loss of TH positive cells and the reduction in dopamine levels were greater in the SNc of rats over-expressing ATF4 than in those over-expressing αS. Moreover, the animals overexpressing ATF4 demonstrated substantial behavior deficits when compared to a control group (Gorbatyuk et al., 2012).

## ATF4 and aging

Age affects the human body by altering proteostasis and enzymatic activity, thereby modulating multiple cellular pathways (Kaushik and Cuervo, 2015). Unfortunately, wide-ranging studies have mainly reported ATF4 over-expression under neurodegenerative conditions, which are prominent in aged tissues and organs. By contrast, ATF4 expression in healthy aged tissues has not received proper attention, with a few exceptions. Our study of aged retinas demonstrated a higher ATF4 expression in retinas from 24-month-old rats than from 4-month-old rats (Lenox et al., 2015). This increase was associated with elevated levels of GADD34 and CHOP proteins. In addition, Rantes/CCL5 expression was enhanced in aged retinas as well. The latest findings for CCL5 support the proposed ATF4 regulation of Rantes secretion through its interaction with the c- Jun molecule of the toll-like receptor 4 (TLR4)- signaling pathway (Zhang et al., 2013). In a further study of naive aged rats, we also demonstrated that 24-month-old rats show significant two-fold elevation of nigral ATF4 levels, which are associated with increases in endogenous α-syn in males and declines in GRP78 in both male and female aged brains (Salganik et al., 2015).

ATF4 has recently been proposed as a potential mediator of age-related muscle weakness and atrophy, as ATF4 downregulation by ursolic acid and tomatidine treatment significantly reduced age- related deficits in skeletal muscle strength, quality, and mass (Ebert et al., 2015). Other studies on age-related muscular dystrophy have revealed that increases in ATF4 expression alone are sufficient to induce fiber atrophy (Ebert et al., 2010). Therefore, ATF4 downregulation could be a potential therapeutic target for restoring skeletal muscle deficit in the elderly.

## Conclusions

In this review, we have presented the latest findings concerning ATF4 involvement in retinal and neurodegenerative disorders. An analysis of the existing literature indicated contradictory opinions on the role of ATF4 in triggering the pro-survival or pro-death trend during developing experimental pathologies. The discrepancy could be due to the specificity of the activated molecular signaling, the stages of neurodegeneration, and the environmental conditions. Moreover, it is noteworthy to mention that the downstream targets and cellular signaling, including autophagy, oxidative stress, inflammation, and translation regulated by ATF4 are quite diverse. However, we believe that the major discrepancy between these studies' results lies in the use of *in vivo* and *in vitro* systems. Importantly, the therapy we are seeking has to define the potential of ATF4 to regulate the progression of neurodegeneration during chronic ER stress, which apparently is difficult, if not impossible, to reproduce in cell-culture disease models. This is also indicated by the fact that most, if not all, published works that have studied the consequences of direct modulation of ATF4 in animal disease models indicate that restriction of the excessive protein production can significantly diminish retinal and neurodegenerative disorders (Sidrauski et al., 2013; Halliday et al., 2015; Rozpedek et al., 2015; Wei et al., 2015; Bhootada et al., 2016; Gully et al., 2016). Therefore, future experiments are necessary to investigate whether downregulation of ATF4 would be a feasible therapeutic strategy for varied neurodegenerative pathologies.

## Author contributions

PP prepared figures, reviewed the literature and prepared the draft. OG reviewed the literature and wrote the review. MG reviewed the literature and wrote the review.

### Conflict of interest statement

The authors declare that the research was conducted in the absence of any commercial or financial relationships that could be construed as a potential conflict of interest.
